# BARD1 recognizes pre-rRNA for DNA damage repair and rRNA biogenesis

**DOI:** 10.1016/j.jbc.2026.111406

**Published:** 2026-03-25

**Authors:** Huang Huang, Duo Wu, Xiaochun Yu

**Affiliations:** 1 State Key Laboratory of Gene Expression, School of Life Sciences, Westlake University, Hangzhou, Zhejiang, China; 2 Westlake Laboratory of Life Sciences and Biomedicine, Hangzhou, Zhejiang, China

**Keywords:** BARD1, BRCT, pre-rRNA binding, homologous recombination, rRNA biogenesis

## Abstract

The Breast cancer susceptibility gene 1 (BRCA1)/BRCA1-associated RING domain 1 (BARD1) complex recognizes pre-ribosomal RNA (pre-rRNA) to coordinate the process of double-strand break (DSB) repair. However, the biological functions and molecular mechanisms under this process have not been explained clearly. Here, we find an important pre-rRNA binding site highly conserved among BARD1 proteins. Mutation of this binding site not only disrupts pre-rRNA binding but also impairs homologous recombination (HR) repair. Moreover, we show that BARD1 localizes in the nucleolus and regulates rRNA biogenesis. Loss of BARD1 reduces levels of pre-rRNA and neosynthesized protein. Collectively, this study reveals that BARD1 plays a crucial role in both DSB repair and protein synthesis.

BRCA1 is a critical tumor suppressor, and BARD1 is a partner of BRCA1 identified by yeast two-hybrid screening. Mutation carriers of the *BRCA1* and *BARD1* genes are predisposed to breast and ovarian cancers (1, 2). The BRCA1/BARD1 complex promotes DNA double-strand break (DSB) repair *via* homologous recombination (HR) and maintains genome integrity. Pathogenic mutations of *BRCA1* or *BARD1* impair DSB repair, leading to chromosomal instability and tumorigenesis (3, 4, 5).

*BRCA1* gene encodes a large nuclear protein with 1863 amino acids that harbors several functional domains (6). The really interesting new gene (RING) domain forms the heterodimer with the RING domain of BARD1, which acts as a ubiquitin E3 ligase (7, 8). The BRCA1 C-terminal (BRCT) domain is a phosphate moiety-binding domain, recognizing not only the protein partners, such as Abraxas, BRCA1-associated C-terminal helicase 1 (Bach1)/Fanconi anemia group J protein (FancJ), and CtBP-interacting protein (CtIP) (9, 10), but also pre-rRNA (11). Mutations within the RING and BRCT domains result in significant structural and functional defects and contribute to an increased risk of carcinogenesis (12, 13, 14). Compared to BRCA1, BARD1 only contains 777 amino acids (15). Similar to BRCA1, BARD1 has an N-terminal RING domain and a C-terminal BRCT domain. Like the BRCA1 BRCT domain, the BARD1 BRCT domain also has a phosphate moiety-binding pocket and is known to recognize pre-rRNA (11, 16). Both BRCA1 and BARD1 are evolutionarily conserved. In particular, the RING domains and the BRCT domains of BRCA1 and BARD1 are conserved in *Caenorhabditis elegans*, suggesting that these functional domains have conserved roles during evolution (17, 18).

Our previous studies show that pre-rRNA is a functional partner of the BRCA1/BARD1 complex (11). Pre-rRNA is transcribed by RNA polymerase I from rDNA loci, extensively processed by numerous enzymes at nucleolus, and shipped to cytoplasm for mature ribosome assembly (19, 20). In response to DSBs, pre-rRNA relocates to DSBs, acting as a scaffold to maintain the DNA damage-induced foci formation (21). Moreover, our earlier studies show that cancer-associated mutations of the *BRCA1* BRCT domain abolish the interaction with pre-rRNA, which impairs the HR repair (11). However, the detailed mechanism by which the BARD1 BRCT domain recognizes pre-rRNA remains elusive.

In addition to the role of pre-rRNA in DSB repair, several lines of evidence suggest that DSB repair factors participate in rRNA biogenesis. It has been shown that loss of FANCA, a key regulator for DNA crosslinking repair, deregulates nucleolar homeostasis, induces mislocalization of key nucleolar proteins, and impairs ribosome assembly (22). Moreover, we have shown that DNA-PK, an important Ser/Thr kinase for non-homologous end joining (NHEJ), phosphorylates a number of nucleolar proteins and facilitates ribosome maturation (23). NBS1, a subunit in the MRN complex, associates with TCOF1 to regulate the pol I-mediated transcription of pre-rRNA (24). Thus, the recent studies reveal a possible crosstalk between DSB repair and rRNA biogenesis.

Here, we further analyze the molecular mechanism by which human BARD1 (HsBARD1) and *Caenorhabditis* *elegan* BARD1 (CeBARD1) bind to pre-rRNA. We show that a conserved arginine residue in HsBARD1 and CeBARD1 mediates the interaction with pre-rRNA. The mutation of this arginine not only impairs HR repair but also rRNA biogenesis, further demonstrating the pivotal role of BARD1 in both biological processes.

## Results

### R640 of CeBARD1 mediates the pre-rRNA binding

**Figure 1:**
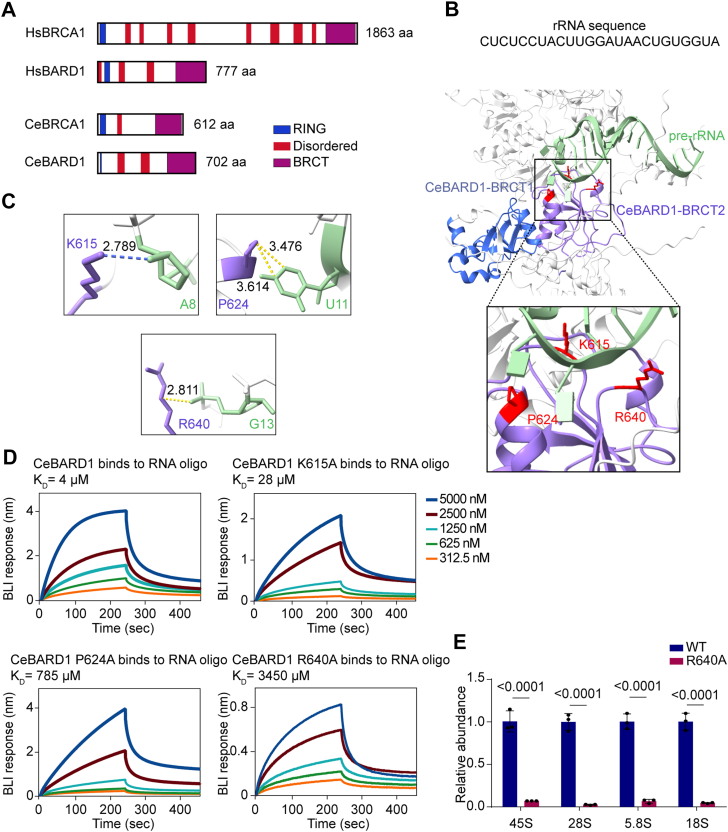
**R640 is essential for the interaction between CeBARD1 and pre-rRNA.***A*, comparative analysis of domain architectures among human and *C. elegans* BRCA1/BARD1 homologs. *B*, identification of pre-rRNA-binding residues through computational prediction. The tandem BRCT domains are rendered with BRCT1 in *blue* and BRCT2 in *purple*. Key residues involved in pre-rRNA binding (K615, P624, and R640) are highlighted in *red*. *C*, close-up view of key interactions at the BARD1 BRCT-RNA interface. *Yellow* dashes indicate non-bonded contacts. *Blue* dashes indicate hydrogen bonds. Distances (Å) are labeled on the dashed lines. *D*, the R640A mutation in CeBARD1 drastically reduces its binding affinity for pre-rRNA. The binding affinities between the CeBARD1 proteins and 25-nt biotin-labeled RNA oligos were measured by BLI assays. *E*, the R640A mutation impairs pre-rRNA binding. The association between the CeBARD1 proteins (residues 1–702; Uniprot #Q21209) and pre-rRNA was examined by protein pull-down and RT-qPCR assays. Data are shown as mean ± SD (n = 3).

While elucidating or predicting the full-length structure of the human BRCA1/BARD1 (HsBRCA1/HsBARD1) complex remains challenging due to extensive flexible regions, the *C. elegans* BRCA1/BARD1 (CeBRCA1/CeBARD1) complex retains the key functional domains and plays a pivotal role in DSB repair (18, 25). Given that CeBRCA1/CeBARD1 possesses significantly fewer flexible linkers and shares high sequence conservation within its core RING and BRCT domains with its human counterpart (Fig. 1*A*), it serves as a tractable model for investigating pre-rRNA binding mechanisms. Human BARD1 harbors two canonical tandem BRCT domains at its C-terminus. Consistently, the *C. elegans* homolog preserves this tandem architecture. Structural superimposition and sequence alignment revealed that the BRCT domains in *C. elegans* are highly homologous to their human counterparts. Notably, the BRCT2 domain displays a particularly high degree of structural conservation (RMSD = 0.948 Å) (Fig. S1).

Thus, to identify the potential binding site between BARD1 and pre-rRNA, we used AF3 to determine the 3D architecture of the CeBRCA1/CeBARD1 (CeBCBD) complex in association with rRNA. We employed full-length *C. elegans* BRCA1 and BARD1 associated with a 25-nt 18S oligonucleotide. This specific RNA sequence was previously identified *via* BARD1 BRCT-capture followed by RNA sequencing as a highly enriched binding motif (11). The resulting AF3 models displayed remarkable structural convergence, with all top-ranked models exhibiting pairwise RMSD values below 1.0 Å (Fig. S2 and Table S1). Prediction results show that residues in the BARD1 BRCT domain (K615, P624, and R640) contribute to RNA binding (Fig. 1, *B* and *C*), which is consistent with our previous finding that BARD1 interacts with pre-rRNA through its BRCT domains (11).

To rigorously assess the specificity of this interaction, we examined the association of BARD1 with highly expressed long non-coding RNAs (lncRNAs). Notably, BARD1 exhibited no detectable binding to 7SK or MALAT1 in pull-down assays coupled with RT-qPCR (Fig. S3, *A* and *B*). This specificity was further corroborated by computational modeling, which demonstrated that the control RNA molecules remained spatially distinct from the canonical RNA-binding pocket of the BARD1 BRCT domain (Fig. S3*C*).

To determine whether the BARD1-RNA interaction is sequence-specific or depends on conserved structural motifs within the pre-rRNA, we generated an additional structural model of the CeBCBD in complex with an alternative 25-nt oligonucleotide derived from the 5.8S rRNA. Remarkably, the AF3-based prediction revealed that this distinct RNA sequence docks at the identical R640 site, closely mirroring the binding mode observed with the 18S rRNA fragment (Fig. S4). Collectively, these results confirm that the BARD1 BRCT domain selectively recognizes pre-rRNA-derived sequences through a conserved structural interface.

Next, to examine the binding affinities between CeBARD1 and pre-rRNA, we performed biolayer interferometry (BLI). CeBARD1 showed a strong affinity with a dissociation constant at 4 μM, while binding affinities of the K615A mutant, the P624A mutant, and the R640A mutant were 28 μM, 785 μM, and 3450 μM, respectively (Fig. 1*D*), suggesting that R640 plays a key role in interaction with RNA. Moreover, we performed a pull-down assay combined with RT-qPCR, the result further confirmed that the R640A mutation disrupted the interaction with different pre-rRNA species (Fig. 1*E*).

### R705 of HsBARD1 plays an important role in the pre-rRNA binding

To delineate the potential binding site between human BARD1 and pre-rRNA, we used AF3 to resolve the 3D architecture of HsBARD1 in association with rRNA. We employed full-length human BARD1 associated with the same 25-nt pre-rRNA oligonucleotide. The resulting AF3 models displayed remarkable structural convergence, with all top-ranked models exhibiting pairwise RMSD values below 1.0 Å (Fig. S5 and Table S2).

Due to the functional importance of R640 in the CeBCBD–pre-rRNA interaction, we sought to identify its corresponding site in humans. Residue R640 of CeBARD1 corresponds to R705 of HsBARD1, and this residue is evolutionarily conserved among all tested species, including human, rat, mouse, and worm (Fig. S6*A*). To explore whether R705 of HsBARD1 also plays a pivotal role in pre-rRNA binding, we performed structural analysis using AF3. The results show that R705 forms two hydrogen bonds with the phosphate backbone of the RNA (Fig. S6, *B* and *C*).

**Figure 2:**
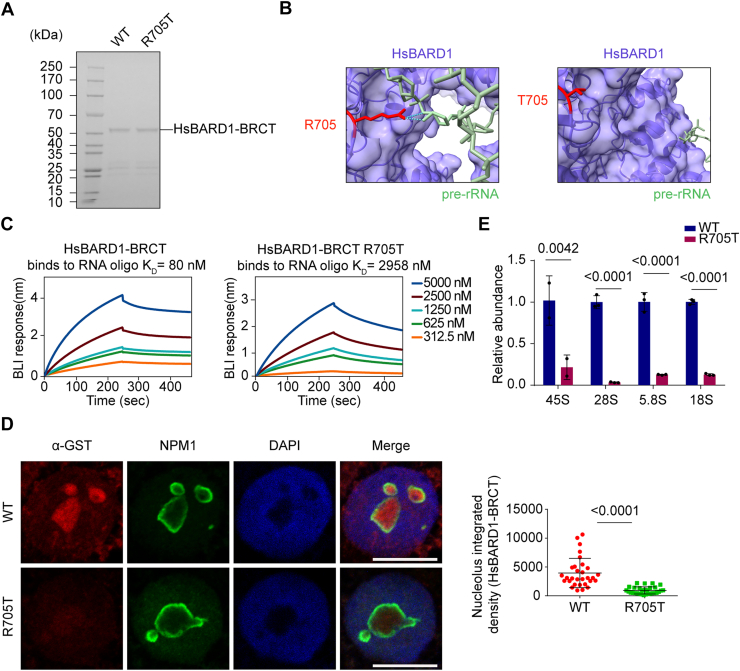
**The R705 residue in human BARD1 is critical for pre-rRNA binding.***A*, SDS-PAGE analysis of purified human BARD1-BRCT protein and the R705T mutant. *B*, AF3-predicted structural models contrast pre-rRNA recognition by wild-type BARD1 (*left*) with the loss of binding in the R705T mutant (*right*). *C*, the R705T mutation in HsBARD1 drastically reduces its binding affinity for pre-RNA. The binding affinities between the HsBARD1-BRCT domains and 25-nt biotin-labeled RNA oligos were measured by BLI assays. *D*, the R705T mutation attenuates BARD1-BRCT aggregation within the nucleolus of U2OS cells. NPM1 was used as a nucleolar marker. The nucleolar density of BARD1 per cell was analyzed (n = 30 cells). Scale bar: 10 μm. *E*, the R705T mutation impairs pre-rRNA binding. The association between the HsBARD1-BRCT domains (residues 554–777; UniProt #Q99728) and pre-rRNA was examined by protein pull-down and RT-qPCR assays. Data are shown as mean ± SD (n = 3).

To examine the biological function of R705 in pre-rRNA binding, we expressed and purified HsBARD1 BRCT and the R705 mutant (Fig. 2*A*). The R705T mutant was chosen based on the OMIM database (a catalog of human genes and genetic disorders) as a variant associated with familial breast cancer but with uncertain significance. AF3 prediction suggests that the R705T mutation abolishes the interaction with pre-rRNA (Fig. 2*B*). Next, we performed BLI assays to validate the AF3 analysis and found that the R705T mutant indeed exhibited a markedly attenuated affinity for pre-rRNA (Fig. 2*C*).

To further validate this interaction, we hybridized fixed cells with recombinant GST-HsBARD1 BRCT and performed IF staining assays. We observed that the recombinant HsBARD1 BRCT domain-bound nucleoli, as indicated by the NPM1 staining as a surrogate marker of nucleolus, where pre-rRNA is transcribed from rDNA loci (26). In contrast, the R705T mutant failed to recognize nucleoli (Fig. 2*D*), suggesting that the mutant loses its ability to bind pre-rRNA *in situ*. We also performed pull-down assays combined with RT-qPCR, further confirming that the R705T mutation disrupted the interaction between the HsBARD1 BRCT domain and pre-rRNA (Fig. 2*E*). These observations indicate that R705 of the HsBARD1 BRCT domain mediates the binding to pre-rRNA.

### The R705T mutation of HsBARD1 impairs the pre-rRNA binding and DNA damage repair

**Figure 3:**
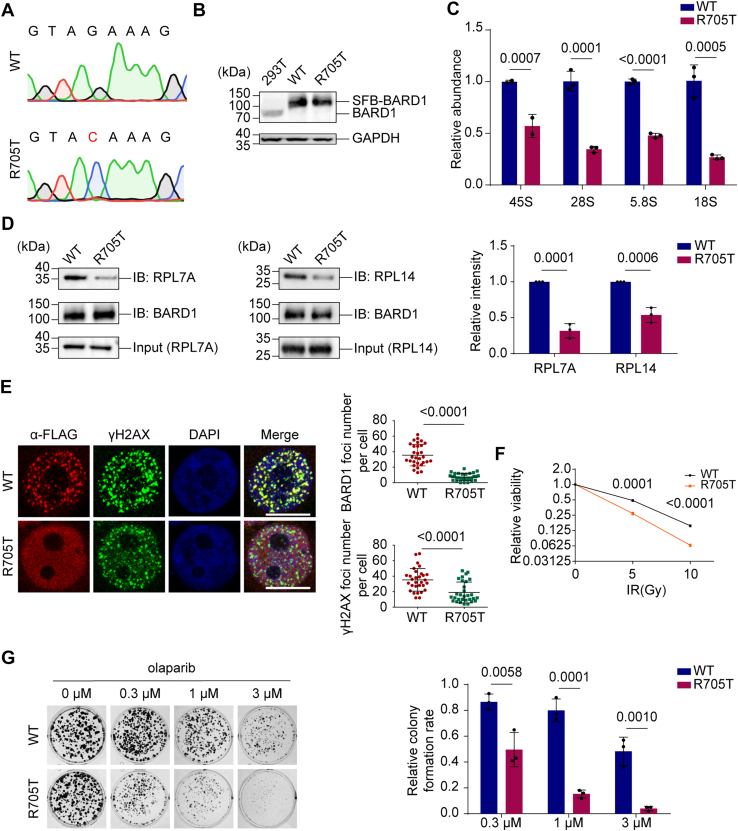
**The R705T mutation impairs pre-rRNA binding and undermines HR repair.***A*, Sanger sequencing chromatograms of 293T cells expressing wild-type BARD1 (*upper*) or the R705T mutant (*lower*). The c.2114G>C substitution is indicated. *B*, immunoblot analysis of lysates from WT 293T cells and their BARD1-KO derivatives. Endogenous BARD1 was ablated *via* CRISPR/Cas9-mediated gene editing, followed by stable reconstitution with either SFB-tagged WT BARD1 or the R705T mutant. This panel validates both the successful depletion of endogenous BARD1 and the comparable expression levels of the exogenous WT and mutant constructs. *C*, the R705T mutation abolishes the association between BARD1 and pre-rRNA. The association was examined by Co-IP assay. Following Co-IP, the enrichment of pre-rRNA was quantified by RT-qPCR. *D*, the R705T mutation abolishes the interactions between BARD1 and ribosomal proteins. Interactions were examined by Co-IP and immunoblotting. *E*, the R705T mutation impairs IRIF of BARD1 and γH2AX. 293T cells expressing wild-type BARD1 or the R705T mutant were treated with 20 Gy of IR. Foci numbers per cell were quantified (n = 30 cells). Scale bar: 10 μm. *F*, the R705T mutation suppresses cell growth. Cells were exposed to the indicated doses of IR. Cell growth was measured with CTG assays. *G*, the R705T mutation sensitizes cells to PARPi treatment. Colony formation was assessed by crystal violet staining. Data are shown as mean ± SD (n = 3).

To determine the functional consequences of the R705T mutation in cells, we established 293T cells stably expressing wild-type (WT) BARD1 and the R705T mutant with the endogenous BARD1 knocked out using the CRISPR/Cas9 system. The genomic DNA was extracted, and successful knockout and mutation were verified by sequencing (Fig. 3*A*). Subsequently, Western blot analysis with an anti-BARD1 antibody confirmed the successful knockout of endogenous BARD1, as only the exogenous FLAG-tagged protein was detected (Fig. 3*B*). The expression levels of wild-type BARD1 and the R705T mutant were comparable (Fig. S7).

Importantly, we confirmed that the R705T mutant maintains the capacity to interact with BRCA1 at levels comparable to WT BARD1 (Fig. S8*A*). It demonstrates that the R705T substitution does not compromise the overall structural integrity of the protein. Interestingly, immunoprecipitation (IP) assays revealed that while WT BARD1 exhibits robust binding to poly (ADP-ribose) (PAR), the R705T mutant exhibits significantly compromised PAR binding (Fig. S8*B*). Given that PAR functions as a structural mimetic of nucleic acids—sharing a repeating phosphate-rich backbone similar to the phosphodiester bonds in RNA—the attenuated affinity observed in the R705T mutant further corroborates the functional impairment of the conserved phosphate-binding pocket within the BRCT domain.

Consistent with the role of R705 in pre-rRNA recognition, cells expressing the R705T mutant showed significantly impaired pre-rRNA binding (Fig. 3*C*). Notably, pre-rRNA-associated proteins, including RPL7A and RPL14, have been shown to localize to DSBs and interact with the BARD1 BRCT domain in an rRNA-dependent manner (11, 27). IP assays revealed that the R705T mutation markedly impaired the interaction between BARD1 and these ribosomal proteins (Fig. 3*D*). To further validate that these protein–protein interactions are mediated by nascent RNA transcripts, we employed BMH21, a small-molecule inhibitor of RNA Polymerase I (Pol I). Pharmacological inhibition of Pol I significantly diminished the association of BARD1 with both pre-rRNA and ribosomal proteins (Fig. S9), thereby substantiating the model that BARD1 directly engages pre-rRNA as a scaffold for complex assembly. These findings underscore the pivotal role of the conserved R705 residue in mediating nucleolar-specific RNA recognition.

Since pre-rRNA directs the BRCA1/BARD1 complex to DSBs, we investigated whether the R705 residue is essential for this targeting process. To this end, following ionizing radiation (IR), cells expressing the R705T mutant showed markedly reduced formation of BARD1 and γH2AX foci (Fig. 3*E*), indicating that the R705T mutation substantially impairs BARD1 recruitment to DSBs.

The BRCA1/BARD1 complex contributes to homologous recombination (HR) repair following its localization to DSBs. Given that HR deficiency increases cellular sensitivity to DNA-damaging agents and PARP inhibitors (PARPis) (28), we found that IR or the PARPi Olaparib significantly suppressed the growth of R705T-mutant cells (Fig. 3, *F* and *G*). These results demonstrate that the R705 residue in BARD1 is indispensable for pre-rRNA binding and, accordingly, for HR-mediated repair of DSBs.

### BARD1 promotes rRNA biogenesis

**Figure 4:**
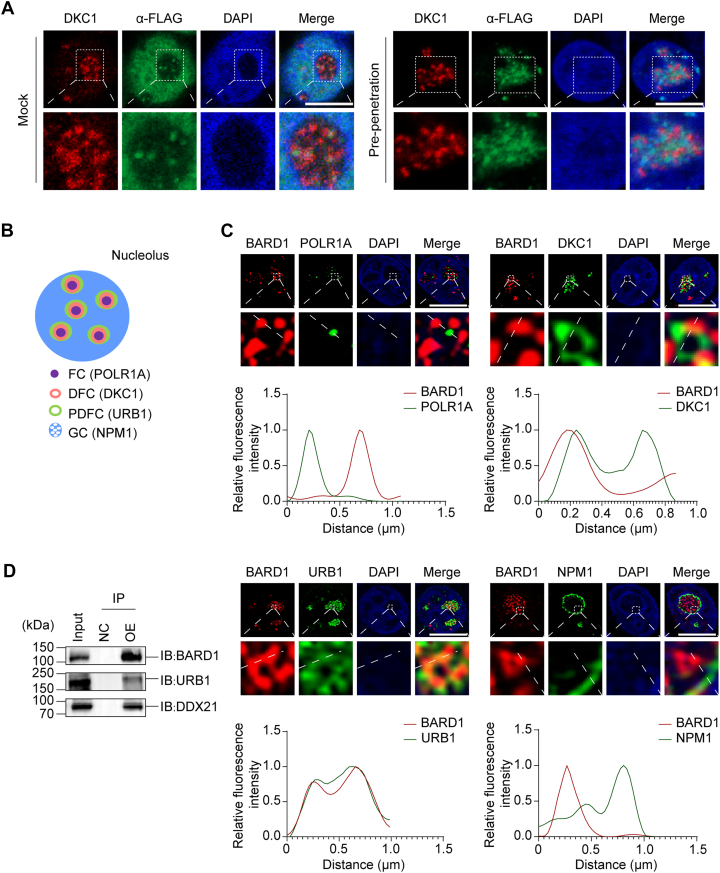
**BARD1 localizes in PDFC.***A*, BARD1 localizes to the nucleolus. The localization of BARD1 in 293T cells was examined by IF with or without detergent pre-treatment. DKC1 was used as a nucleolar marker. Scale bar, 10 μm. *B*, a schematic illustration depicts the distinct sub-regions of the nucleolus. POLR1A, DKC1, URB1, and NPM1 are specific markers for FC, DFC, PDFC, and GC, respectively. *C*, BARD1 is enriched in PDFC. FLAG-BARD1 and GFP-URB1 were expressed in 293T cells. Representative SIM images of BARD1 and markers of FC, DFC, PDFC, and GC were acquired. The fluorescence intensity along the white dashed line was measured (*lower panels*). Scale bar, 10 μm. *D*, BARD1 interacts with proteins in PDFC. Co-IP and immunoblotting were performed in 293T cells overexpressing SFB-NC or SFB-BARD1.

Interestingly, in addition to its role in the DNA damage response, we found that BARD1 also accumulates in the nucleolus. For clear visualization, cells were pre-treated with detergent before fixation, which revealed prominent nucleolar localization of BARD1 under non-stressed conditions (Fig. 4*A*), suggesting functions beyond DNA repair.

The nucleolus is the primary site for rRNA biogenesis and is organized into distinct subregions: the fibrillar center (FC) for pre-rRNA transcription, the dense fibrillar component (DFC) and periphery of DFC (PDFC) for pre-rRNA processing, and the granular component (GC) for ribosome assembly (Fig. 4*B*) (29, 30, 31). To investigate BARD1's nucleolar function, we performed structured illumination microscopy (SIM) using established subnucleolar markers: POLR1A (FC), DKC1 (DFC), URB1 (PDFC), and NPM1 (GC). BARD1 showed predominant colocalization with URB1 (Fig. 4*C*). Co-IP assays further confirmed BARD1's interaction with PDFC-resident proteins (Fig. 4*D*) (32). Notably, this interaction was sensitive to RNase treatment and was abolished by the R705T mutation, demonstrating that the recruitment of BARD1 to the PDFC is strictly RNA-dependent (Fig. S10). Collectively, these data indicate that BARD1 primarily localizes to the PDFC subdomain.

**Figure 5:**
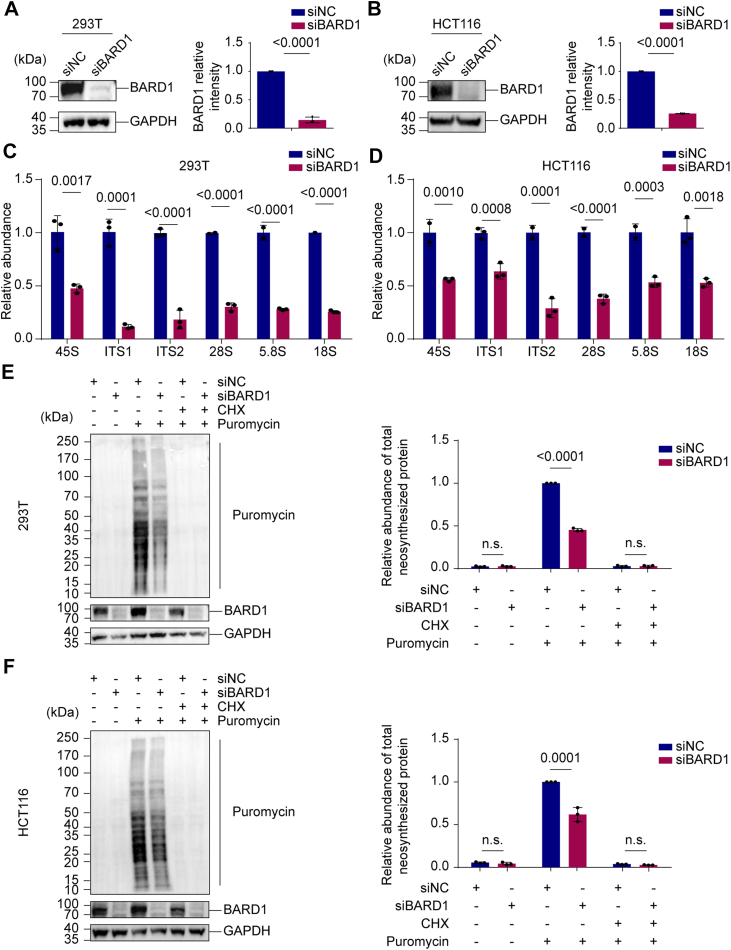
**BARD1 regulates rRNA biogenesis.***A*, siRNA targeting *BARD1* reduces the protein levels of BARD1 in 293T cells. *B*, siRNA targeting *BARD1* reduces the protein levels of BARD1 in HCT116 cells. *C*, loss of BARD1 impairs pre-rRNA biogenesis. RT-qPCR was performed to detect the precursor, intermediate, and mature rRNA in 293T cells. *D*, loss of BARD1 impairs pre-rRNA biogenesis. RT-qPCR was performed to detect the precursor, intermediate, and mature rRNA in HCT116 cells. *E*, protein synthesis is suppressed by *BARD1* siRNA knockdown in 293T cells. The newly synthesized protein levels were measured by puromycin incorporation assays. CHX treatment served as a positive control. *F, p*rotein synthesis is suppressed by *BARD1* siRNA knockdown in HCT116 cells. The newly synthesized protein levels were measured by puromycin incorporation assays. CHX treatment served as a positive control. Data are shown as mean ± SD (n = 3).

Given that PDFC proteins form a pericentral shell around FC/DFC units to support RNA polymerase I processivity by providing a fluid microenvironment, we hypothesized that BARD1 contributes to rRNA biogenesis. siRNA-mediated knockdown of BARD1 in different cell lines (Fig. S11; Fig. 5, *A* and *B*) significantly reduced the levels of pre-rRNA (45S), internal transcribed spacers (ITS1, ITS2), and mature rRNAs (28S, 5.8S, 18S) (Fig. 5, *C* and *D*). Given the central role of rRNA in ribosome function, we assessed global translation and observed a marked decrease in nascent protein synthesis in BARD1-depleted cells (Fig. 5, *E* and *F*).

To further establish the biological imperativeness of the BARD1–pre-rRNA interaction, we performed stringent functional complementation assays. Re-expression of WT BARD1 in BARD1-depleted cells effectively rescued the pre-rRNA biogenesis defects. In contrast, the RNA-binding-deficient R705T mutant failed to restore rRNA processing (Fig. S12*A*). Furthermore, WT BARD1, but not the R705T mutant, successfully restored global protein synthesis levels, as assessed by puromycin incorporation assays (Fig. S12*B*). Collectively, these findings demonstrate that the specific recognition of pre-rRNA by BARD1 is an essential prerequisite for efficient rRNA biogenesis and downstream translational output.

**Figure 6:**
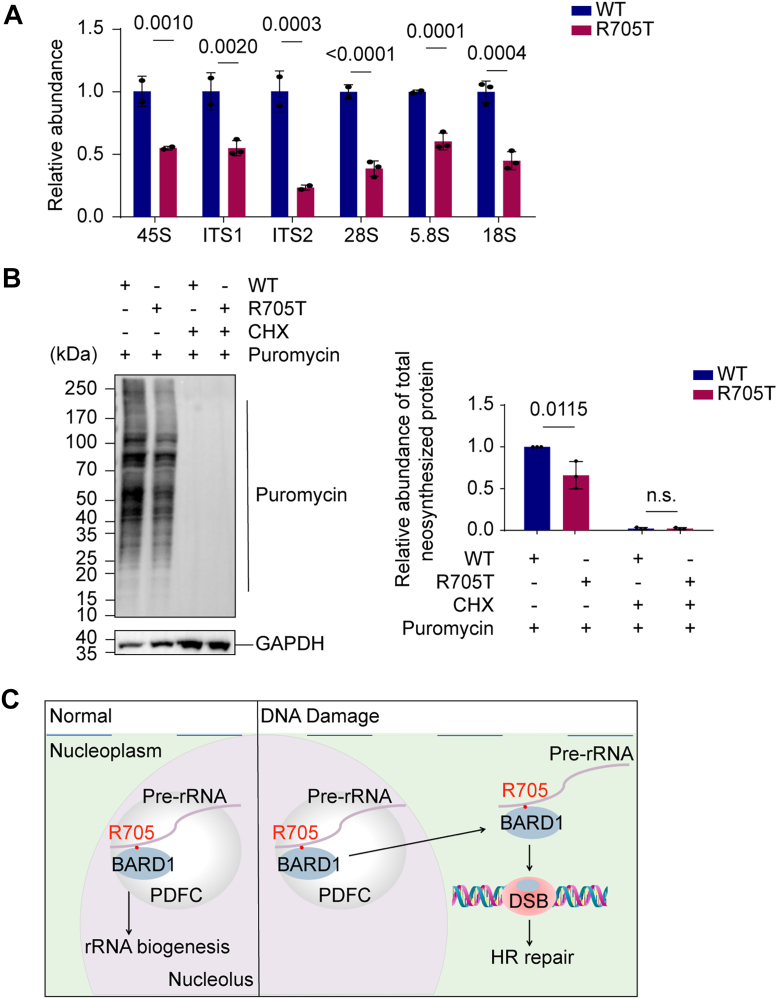
**The R705T mutation affects rRNA biogenesis.***A*, the R705T mutation impairs pre-rRNA biogenesis. RT-qPCR was performed to detect the precursor, intermediate, and mature rRNA. *B*, protein synthesis is suppressed in cells expressing the R705T mutant. The newly synthesized protein levels were measured by puromycin incorporation assays. CHX treatment served as a positive control. *C*, a working model depicting how pre-rRNA binding orchestrates the dual functions of BARD1 in DNA repair and rRNA biogenesis. Data are shown as mean ± SD (n = 3).

Notably, the R705T mutant cells displayed impaired rRNA biogenesis and reduced translational capacity (Fig. 6, *A* and *B*). Together, these findings demonstrate that pre-rRNA recognition *via* the R705 residue is essential for BARD1's nucleolar targeting and its role in ribosomal RNA synthesis.

## Discussion

In this study, we identify a conserved arginine residue (R640) within the BRCT domain of *C. elegans* BARD1 that is indispensable for pre-rRNA binding. The orthologous mutation in human BARD1 (R705T) similarly abrogates interactions with pre-rRNA and its associated protein machinery. These findings align with established principles of protein-RNA recognition, where arginine side chains serve as critical mediators by enhancing binding affinity through electrostatic interactions and hydrogen bonding, while conferring sequence specificity (33, 34). Importantly, while the clinical C645R mutation in human BARD1 destabilizes the BRCT fold and indirectly impairs RNA binding (11, 16), substitution of R640 in *C. elegans* or R705 in human BARD1 with threonine preserves the structural integrity and heterodimerization capacity of the BRCT domain (Figs. S8*A* and S13). Thus, R705 represents a *bona fide* surface-exposed residue that directly orchestrates pre-rRNA recognition.

Mechanistically, AF3 modeling provides atomistic insight into this interface, revealing a conserved recognition mode where BARD1 anchors a consensus footprint (5′-CUCUCCUACUUGGAUAA-3′). This structural model is strongly corroborated by our biochemical data, which demonstrate that BARD1 affinity is both length- and sequence-dependent (11). Stereochemically, the small van der Waals radii and high conformational flexibility of pyrimidines (C/U) allow the motif to navigate the sterically restricted BRCT binding pocket—a geometry that excludes bulky polypurine tracts. This suggests an evolutionary optimization, where a pyrimidine "key" facilitates pocket entry, while flanking purine "anchors" (*e.g.*, GCA/GGA) provide interfacial stability through π−π stacking. The high specificity of this interaction is further underscored by BARD1’s inability to bind abundant non-cognate transcripts like 7SK and MALAT1, which lack this consensus signature (Fig. S3). Remarkably, we found that an identical 10-nt segment (CGCACUUGCG) is shared between the 18S and 5.8S rRNA fragments, both of which recapitulate the same binding mode (Fig. S4). This consistency across distinct rRNA species underscores the high specificity and universal nature of the BARD1–pre-rRNA interaction.

PAR is a transient PTM induced by genomic stress that mediates the instantaneous recruitment (priming) of numerous DNA repair factors to the proximity of DNA lesions (35). However, it is also quickly hydrolyzed by PARG to unload repair factors to the lesions for DNA repair. We hypothesize a "handover mechanism" wherein pre-rRNA subsequently "replaces" PAR, acting as a stable molecular tether that ensures the sustained retention of the complex at lesions. Substantial evidence indicates that this pre-rRNA-mediated tethering is essential for facilitating HR repair. Accordingly, we found that the R705T mutation sensitizes cells to DNA-damaging agents and PARP inhibitors, mirroring the HR deficiency reported for R705A mutants (36). It is highly probable that the R705T mutant fails this critical "handover," leading to the premature dissociation of BARD1 from DSBs and the subsequent failure of downstream repair steps.

Beyond its role in DNA repair, we demonstrate that the R705 residue is critical for BARD1-mediated rRNA biogenesis. Under basal conditions, BARD1 localizes to the nucleolus, where its depletion or mutation (R705T) compromises pre-rRNA transcription and processing, leading to reduced mature rRNA levels and suppressed protein synthesis. Notably, although the R705T mutation impairs the affinity of BARD1 for PAR, PARP activity itself is dispensable for basal rRNA biogenesis (23, 37). The observation that pharmacological inhibition of PARP fails to phenocopy the severe rRNA processing defects seen in R705T cells underscores that the BARD1–pre-rRNA interface, anchored by R705, serves as the primary functional driver within the nucleolus. And to rule out the possibility that reduced binding in the R705T mutant is merely a secondary effect of impaired rRNA biogenesis, we performed all *in vitro* binding and pull-down assays using fixed, equal concentrations of purified protein and RNA. Under these normalized conditions, the reduced binding of R705T reflects an intrinsic loss of affinity, independent of cellular rRNA levels. Although the BRCA1/BARD1 complex primarily functions as an E3 ubiquitin ligase, BARD1 alone lacks catalytic activity (38). For instance, BARD1 enhances the helicase activity of BLM (39), a protein that may also localize to the nucleolus and participates in pre-rRNA processing by unwinding newly transcribed pre-rRNA (40, 41, 42). Thus, BARD1 may mechanistically scaffold BLM to facilitate rRNA maturation. Furthermore, given the capacity of BARD1 and pre-rRNA to undergo liquid–liquid phase separation (LLPS) (11), we hypothesize that RNA binding maintains the biophysical properties of BARD1-enriched condensates within the nucleolar subdomains, thereby optimizing the environment for rRNA production. Notably, BRCA1, the primary binding partner of BARD1, has been shown to regulate rRNA synthesis by interacting with RNA polymerase I machinery (43) and modulating the expression of ribosomal proteins (44, 45), suggesting potential synergy between BRCA1 and BARD1 in supporting rRNA biogenesis.

In summary, our work delineates a conserved molecular mechanism through which BARD1 engages pre-rRNA *via* a surface-exposed arginine residue within its BRCT domain (Fig. 6*C*). This interaction is essential not only for DSB repair but also for nucleolar localization and rRNA biogenesis, revealing a dual functionality for the BARD1–pre-rRNA interface in safeguarding genomic integrity and supporting cellular translational capacity.

## Experimental procedures

### Antibodies and reagents

Anti-FLAG (rabbit, catalog no.: F7425; mouse, catalog no.: F1804) and anti-puromycin (mouse, catalog no.: ZMS1016) antibodies were purchased from Sigma. Anti-GST (mouse, catalog no.: 2624S) antibody was purchased from Cell Signaling Technology. Anti-BARD1 (rabbit, catalog no.: NB100-319) and anti-γH2AX (rabbit, catalog no.: NB100-384) antibodies were purchased from Novus. Anti-RPL7A (rabbit, catalog no.: A13713) antibody was purchased from ABclonal. Anti-RPL14 (rabbit, catalog no.: 14991-1-AP), anti-NPM1 (mouse, catalog no.: 60096-1-Ig), anti-URB1 (rabbit, catalog no.: 20023-1-AP), and anti-DDX21 (rabbit, catalog no.: 10528-1-AP) antibodies were purchased from Proteintech. Anti-POLR1A (mouse, catalog no.: sc-48385) and anti-DKC1 (mouse, catalog no.: sc-373956) antibodies were purchased from Santa Cruz Biotechnology. Anti-GFP (rabbit, catalog no.: A-21311) antibody was purchased from Thermo Fisher Scientific. Anti-BRCA1 (mouse, catalog no.: ab16780) antibody was purchased from Abcam. Anti-PAR (mouse, catalog no.: 4335-MC-100) antibody was purchased from R&D. Anti-GAPDH (Rabbit, catalog no.: ET1601-4) antibody was purchased from HUABIO. Puromycin (60210ES25), Olaparib (S1060), and Cycloheximide (HY-12320) were purchased from YEASEN, Selleck, and MCE, respectively.

### Plasmid constructs

CeBARD1 (full-length, 702aa) was cloned into the pET-30a vector, with an N-terminal His6 tag, the mutations (K615A, P624A, and R640A) were generated using the Fast Mutagenesis Kit (Vazyme, C214-01). HsBARD1-BRCT (residues 554-777aa) was cloned into pGEX-4-T1, with an N-terminal GST tag, and the mutation (R705T) was generated using the Fast Mutagenesis Kit (Vazyme, C214-01). HsBARD1 (full-length, 777aa) was cloned into the SFB-puro vector, with an N-terminal FLAG tag, and the mutation (R705T) was generated using the Fast Mutagenesis Kit (Vazyme, C214-01). Enhanced GFP (EGFP) was added to the N-terminus of URB1. The primers are listed in Table S3.

### Protein expression and purification

All proteins were produced in *Escherichia coli* cells (BL21). Bacterial cells were grown at 37 °C to an OD600 of approximately 0.6, then proteins were induced with 0.5 mM IPTG at 16 °C overnight. Bacterial cells were collected by centrifugation at 8000 rpm for 10 min. Cells expressing GST tag proteins were lysed with NETN100 (0.05% NP40, 20 mM Tris–HCl, pH 8.0, 100 mM NaCl, 1 mM EDTA, 1 mM PMSF), after sonication and centrifugation, clear lysate were collected and incubated with glutathione Sepharose (Cytiva, 17075605) at 4 °C for 2 h, the beads were washed with NETN wash buffer (20 mM Tris–HCl, pH 8.0, 100 mM NaCl) three times, GST tag proteins were then eluted with 20 mM L-glutathione reduced (Sigma, G4251), and dialyzed in buffer (1×PBS) at 4 °C overnight. Cells expressing His6 tag proteins were lysed with His resuspension buffer (25 mM Tris–HCl, pH 7.0, 500 mM Nacl, 2 mM 2-mercaptoethanol, 5% glycerol, 1 mM PMSF), after sonication and centrifugation, clear lysate were collected and incubated with Ni Sepharose (Cytiva, 17531802) at 4 °C for 2 h, washed with His wash buffer (50 mM imidazole in resuspension buffer), then eluted with 250 mM imidazole, and dialyzed at 4 °C overnight in buffer (1×PBS).

### Biolayer interferometry (BLI) assay

Interactions between proteins and RNA were measured by Octet Red96e (Sartorius). 5′-biotinylated RNA oligo (5′-AUCGACACUUCGAACGCACUUGCGG-3′) was synthesized by Tsingke Biotechnology Co., Ltd. Streptavidin biosensors were first balanced with kinetics buffer (1X PBS containing 0.05% Tween-20) for 1 min, followed by PBS buffer washing. The biosensor was then incubated in kinetics buffer containing gradient concentrations of RNA for 4 min to allow association, and dissociation for 4 min. Biosensors loaded with RNA but without adding proteins act as controls. Binding affinities were calculated using Octet Data Analysis HT 12.0 software, reference signals were subtracted with the Y axis aligned to baseline, inter-step correction aligned to dissociation, then analyzed in a global 1:1 fit model.

### RNA and protein pull-down assay

To investigate the direct interaction between BARD1 and RNA, recombinant GST-tagged or His-tagged proteins (5 μg) were incubated with total RNA (5 μg) extracted from HeLa cells in the presence of appropriate affinity resin (Glutathione Sepharose or Ni-NTA beads). Reactions were conducted at 4 °C for 2 h with gentle rotation to equilibrate the binding complex. Following incubation, the protein-bound beads were subjected to three stringent washes with wash buffer to minimize non-specific background associations. Bead-associated RNA was then isolated using TRIzol reagent according to the manufacturer's protocol. The purified RNA was subsequently reverse-transcribed into complementary DNA (cDNA) using the PrimeScript II first Strand cDNA Synthesis Kit (Takara, 6210A). Quantitative PCR (qPCR) was performed using the qPCR SYBR Green Master Mix (Yeasen, 11184ES08). Relative RNA levels were calculated using the 2^∧-ΔΔCt^ method. Specific primers are listed in Table S4.

### Western blotting

Proteins were denatured at 98 °C for 5 min, separated by electrophoresis on 4 to 20% SDS-PAGE gels, and subsequently transferred onto 0.45 μm PVDF membranes (Sigma, IPVH00010). The membranes were blocked with 3% BSA for 30 min and then incubated with primary antibodies (1:1000 dilution) for 1 h at room temperature. After three washes with TBST, the membranes were incubated with an HRP-conjugated secondary antibody for 1 h. Protein bands were visualized using an ECL detection reagent (Thermo, 34580).

### Immunofluorescence (IF) staining

For foci analyzing, cells were seeded on 6 well-plate, after 24 h, the plate was irradiated with X-ray biological irradiator (Rad Source RS2000 Pro) at dose of 20 Gy, after recover at 37 °C with 5% CO_2_ overnight, cells were fixed with 4% PFA for 15 min, washed with PBS twice, then followed by permeabilization with 0.5% Triton X-100 for 7 min. Cells were incubated with the primary antibodies (1:1000 dilution) for 1 h. Coverslips were washed with PBS three times, incubated with secondary antibodies for 1 h, and stained with DAPI. The images were captured by Zeiss 900 at ×63.

For GST fusion protein binding, cells were seeded on a 6-well plate. After 24 h, the cells were washed with PBS containing RNase inhibitor (1:1000), permeabilized with 0.5% Triton X-100 for 7 min, and fixation with 4% PFA for 15 min. Cells were incubated with the GST fusion protein at 37 °C for 1 h before primary antibody incubation. After being washed with PBS three times, cells were incubated with secondary antibodies for 1h and stained with DAPI (Yeasen, 36308ES20). The images were captured by Zeiss 900 at ×63.

### Cell culture

293T, HCT116, and U2OS cells were purchased from American Type Culture Collection. U2OS cells were cultured in U2OS special medium (Pricella, CM-0236) containing McCoy’s 5A, 10% fetal bovine serum (FBS), and 1% penicillin-streptomycin(P/S) solution. 293T and HCT116 cells were cultured in DMEM (Gibco, C11995500BT) supplemented with 10% FBS (ExCell Bio, FND500) and 1% P/S. All cells were grown in a humidified incubator at 37 °C with 5% CO_2_.

### Stable cell line construction

We established two stable cell lines. The first is the WT BARD1 (with four synonymous mutation sites) expression cell line, and the other is the R705T BARD1 (R705T mutation upon synonymous mutation) cell line. sgRNAs targeting *HsBARD1* were designed at CRISPick (https://portals.broadinstitute.org/gppx/crispick/public). Four candidate sequences were picked, PAM sequence recognized by sgRNAs or sequence near PAM were selected to do synonymous mutation, HsBARD1 overexpression plasmids (WT and R705T) with synonymous mutations were transfected into 293T cells by lipo3000 (Invitrogen, 290459), 2.5 μg/ml puromycin was added for 48 h to select overexpression cells, then cells inoculated to 96-well plate at dilution ratio of 1:10,1:100, and 1:1000 to get single cells. WB and IF were used to detect the effect of overexpression.

sgRNA containing scaffold (EGFP tag, PX330-based plasmids) was transfected into the overexpression cells by lipo3000 to knock out endogenous HsBARD1. After 48 h, single cells expressing EGFP were sorted by Flow Cytometry (BD, FACS Aria Fusion SORP) and seeded into a 96-well plate (one single cell/well) to grow. Synonymous mutation sites are listed in Table S5. sgRNA sequences are listed in Table S6.

### Immunoprecipitation (IP) assay

Stable cell lines expressing WT BARD1 or mutant BARD1 (R705T) were lysed with NETN420 buffer containing protein inhibitor (Beyotime, P1011-1, 1:1000) and RNase inhibitor at 4 °C for 15 min, supernatant was collected and incubated with streptavidin agarose resin (Thermo, 20353) at 4 °C for 2 h. Beads were centrifuged and washed with NETN100 three times. Samples were used to do WB or RT-qPCR.

### RNA interference

siRNA targeting *BARD1* was purchased from Tsingke Biotechnology Co., Ltd. Transient depletion of BARD1 was carried out using Lipofectamine RNAiMAX (Invitrogen, 13778075) according to the manufacturer’s instructions. The sequences of the siRNAs are listed in Table S7.

### Cell viability assay

Cells were seeded in 96-well plates. After 24 h, cells were treated with Olaparib. After incubation for 6 days, cell viability was measured using the CellTiter-Glo reagent (Promega, G9242) according to the manufacturer's instructions.

### Colony formation assay

Cells were seeded on 6-well plates. After 24 h, cells were treated with Olaparib. After incubation for 14 days, clone formation was measured using 1% crystal violet.

### Structured illumination microscopy (SIM) procedure

Cells were seeded in cellvis dishes. After 24 h, cells were washed with PBS, followed by permeabilization with 0.5% Triton X-100 and fixed with 4% PFA. Then, the cells were blocked in 5% BSA for 1 h and hybridized with the primary antibodies (1:200 dilution) overnight. After being washed with TBST, the cells were incubated with secondary antibodies and DAPI. Then the dishes were checked with the multi-modal structured light super-resolution microscopy system (NanoInsights-Tech Co., Ltd), and images were captured with a 3D-SIM model. Then the SIM Imaging Analyser software (NanoInsights-Tech) was adopted for image reconstruction.

### Puromycin incorporation assay

Cells were pre-treated with DMSO or 50 μg/ml CHX for 2 h. Then, the indicated cells were incubated with 10 μg/ml puromycin for 10 min. Cell extracts were used for WB.

### Statistical analysis

All results were analyzed using GraphPad Prism 10.0 software. Data were presented as mean ± SD. Statistical significance was subjected to Student’s two-tailed *t* test. *p* < 0.05 was considered statistically significant.

## Data availability

All data supporting the findings of this study are available within the paper and its supporting information.

## Supporting information

This article contains supporting information.

## Conflict of interest

The authors declare that they have no conflicts of interest with the contents of this article.
