# Supplementary material for: BARD1 recognizes pre-rRNA for DNA damage repair and rRNA biogenesis
Source: J Biol Chem. 2026 Mar 25;302(5):111406. doi: 10.1016/j.jbc.2026.111406 (PMC13098419; doi:10.1016/j.jbc.2026.111406)
Supplement: Supporting Figures and Tables [file mmc1.pdf]

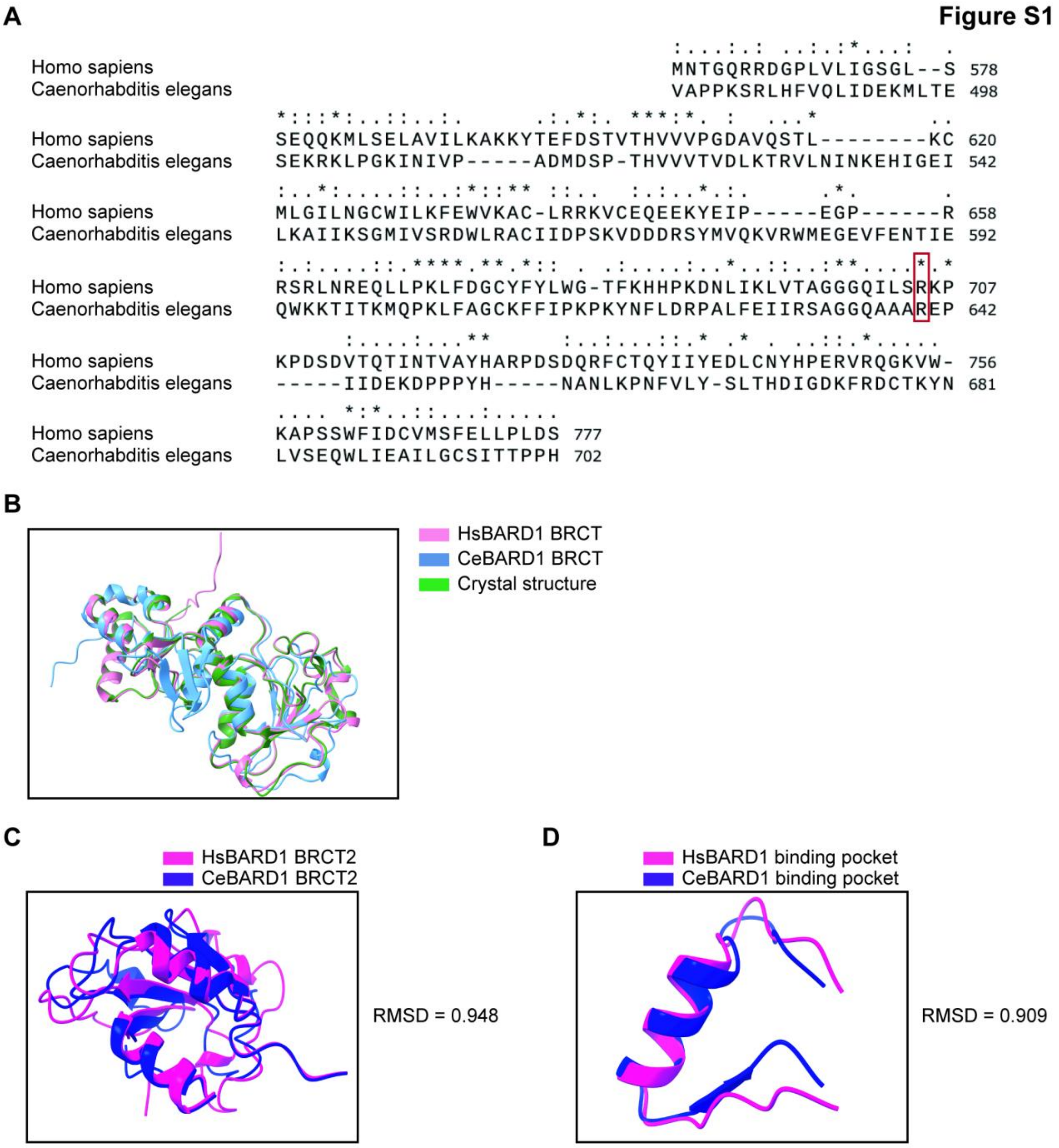

Figure S1. Evolutionary conservation and structural homology of BARD1 BRCT domains.

(A) Sequence alignment of the tandem BRCT domains. The human BARD1 BRCT (residues 560–777; UniProt Q99728) is aligned with its *C. elegans* ortholog (residues 499–702; UniProt Q21209). Sequence conservation is annotated: asterisks (*) denote invariant residues, colons (:) indicate strong physicochemical conservation, and periods (.) signify moderate conservation. Notably, the arginine residue at position 640 (R640, red box) is evolutionarily invariant (conservation score = 100).

(B) Structural superposition of tandem BARD1 BRCT domains. Structural superposition of the *C. elegan*s tandem BRCT domains (AF3-predicted) with the human BARD1 counterpart, including both an AF3-predicted model and a high-resolution crystal structure (PDB ID: 2NTE). The *C. elegans* architecture exhibits a remarkable degree of structural homology to the human domains. The high modeling accuracy is reflected by an RMSD of 0.456 Å between the AF3-predicted human model and the corresponding crystal structure. Notably, the cross-species structural alignment between the *C. elegans* and human tandem BRCT domains yields an RMSD of 1.176 Å. (C) Structural homology of the BRCT2 domains. The second BRCT domain (BRCT2) from *C. elegans* (residues 601–702) and human BARD1 (residues 667–777) demonstrates high topological conservation. The calculated RMSD value (Å) is indicated in the panel. (D) Structural conservation of the putative RNA-binding pocket. Superposition of the potential pre-rRNA binding cleft (residues 615–640 in *C. elegans*) against the orthologous human BARD1 ligand-binding pocket. The highly conserved spatial arrangement of the residues within this pocket, including the invariant R640 anchor, is characterized by a low RMSD value (Å) as determined via UCSF ChimeraX.

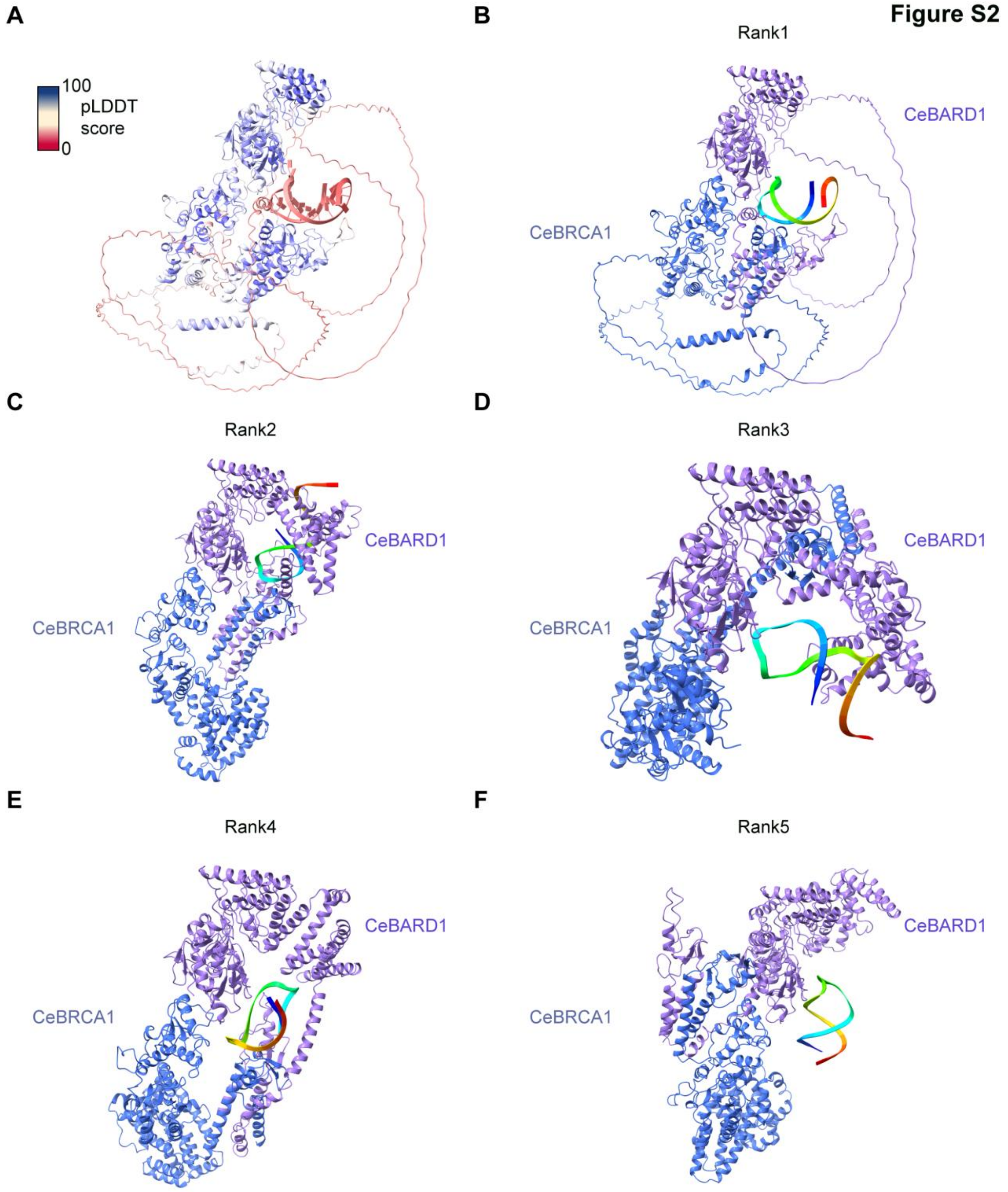

Figure S2. AlphaFold-Multimer analysis of the CeBCBD–pre-rRNA complex.

(A) Structural model of the *C. elegans* complex colored by per-residue pLDDT scores, blue and red denote high and low prediction confidence, respectively. (B–F) Representation of the top five ranked structural models generated by AF3 at a 1:1 stoichiometry. Pairwise RMSD values between all models remain below 1.0 Å, indicating high structural convergence. Observed variations are primarily localized to conformational flexibility within intrinsically disordered regions.

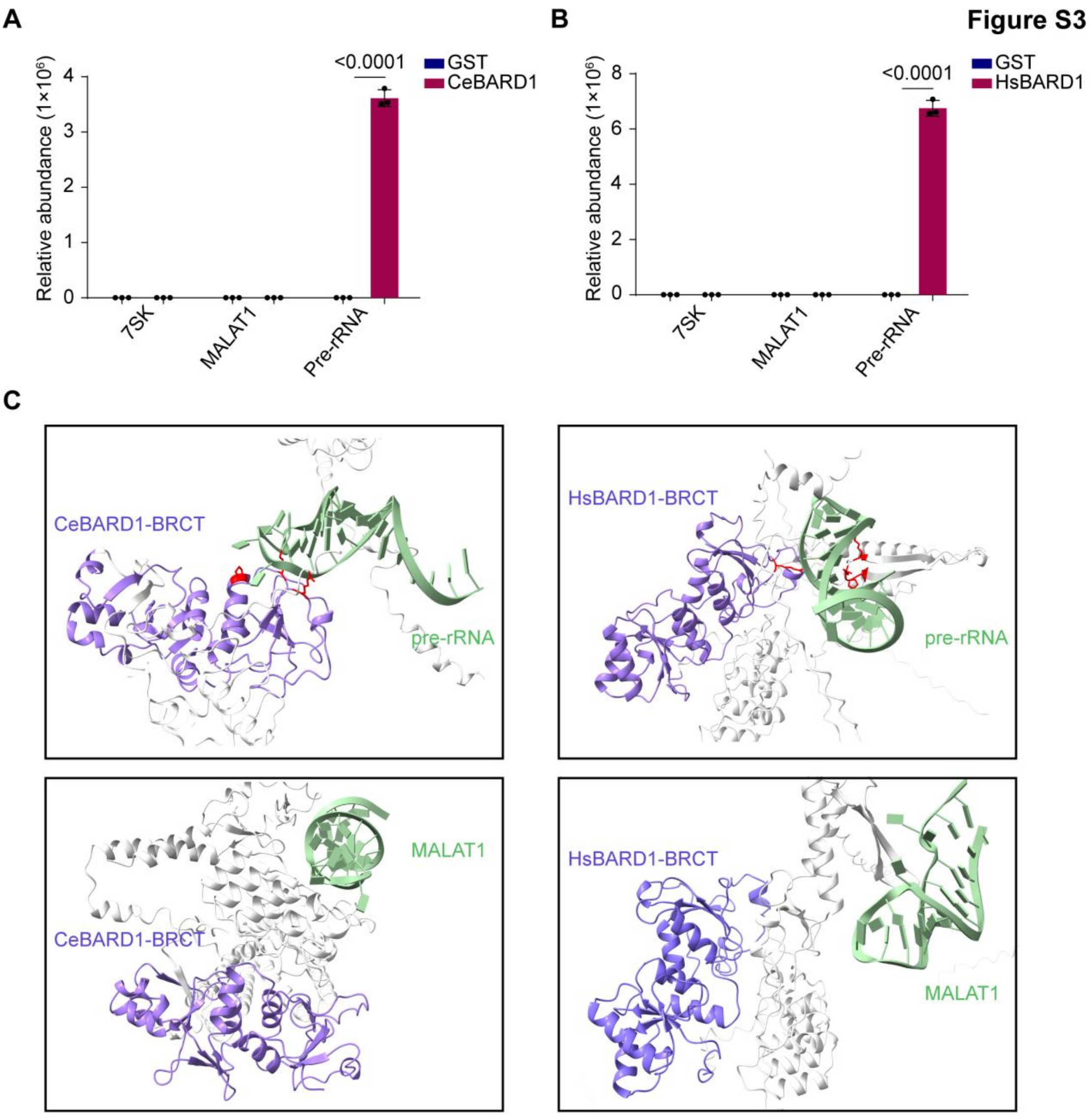

Figure S3. BARD1 exhibits intrinsic binding specificity for pre-rRNA.

(A–B) Selective association of CeBARD1 (A) and HsBARD1 (B) with pre-rRNA. Pull-down assays followed by RT-qPCR show significant enrichment of pre-rRNA species, whereas negligible binding is observed for the lncRNAs MALAT1 and 7SK. (C) Structural modeling of BARD1–RNA interaction specificity via AF3. AF3-predicted models illustrate the interaction between BARD1 (CeBARD1, left; HsBARD1, right) and distinct RNA ligands. Upper panels: Cognate pre-rRNA oligonucleotides bind with the BRCT domains. Lower panels: Non-cognate 25-nt oligonucleotides randomly derived from MALAT1 are positioned distally to the

functional interface. Key residues involved in pre-rRNA binding are highlighted in red. This spatial segregation provides a distinct structural contrast to the specific pre-rRNA binding mode. Data are shown as mean ± SD (n = 3).

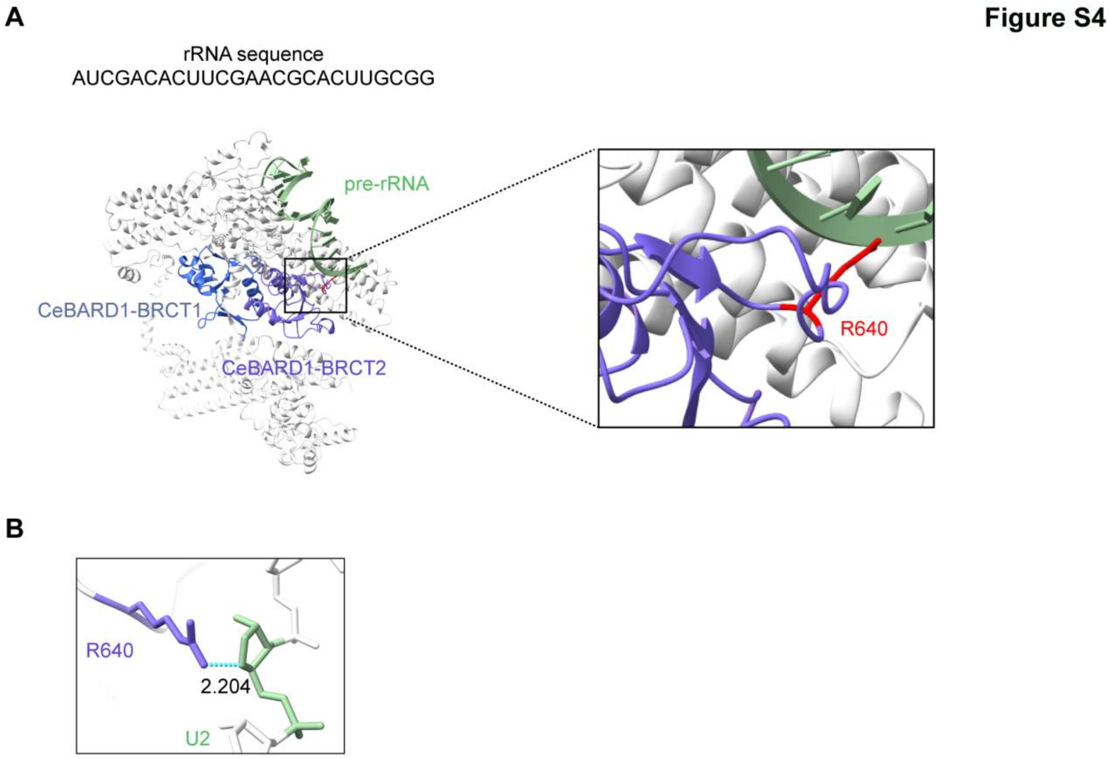

Figure S4. Conserved binding mode of CeBCBD across different rRNA species.

(A) Computational identification of residues involved in 5.8S rRNA binding. The critical residue R640 is highlighted in red, demonstrating a binding site consistent with that used for 18S rRNA. (B) Close-up view of the *C. elegans* BARD1 BRCT–RNA interface. Hydrogen bonds are indicated by blue dashes, with distances (Å) specified.

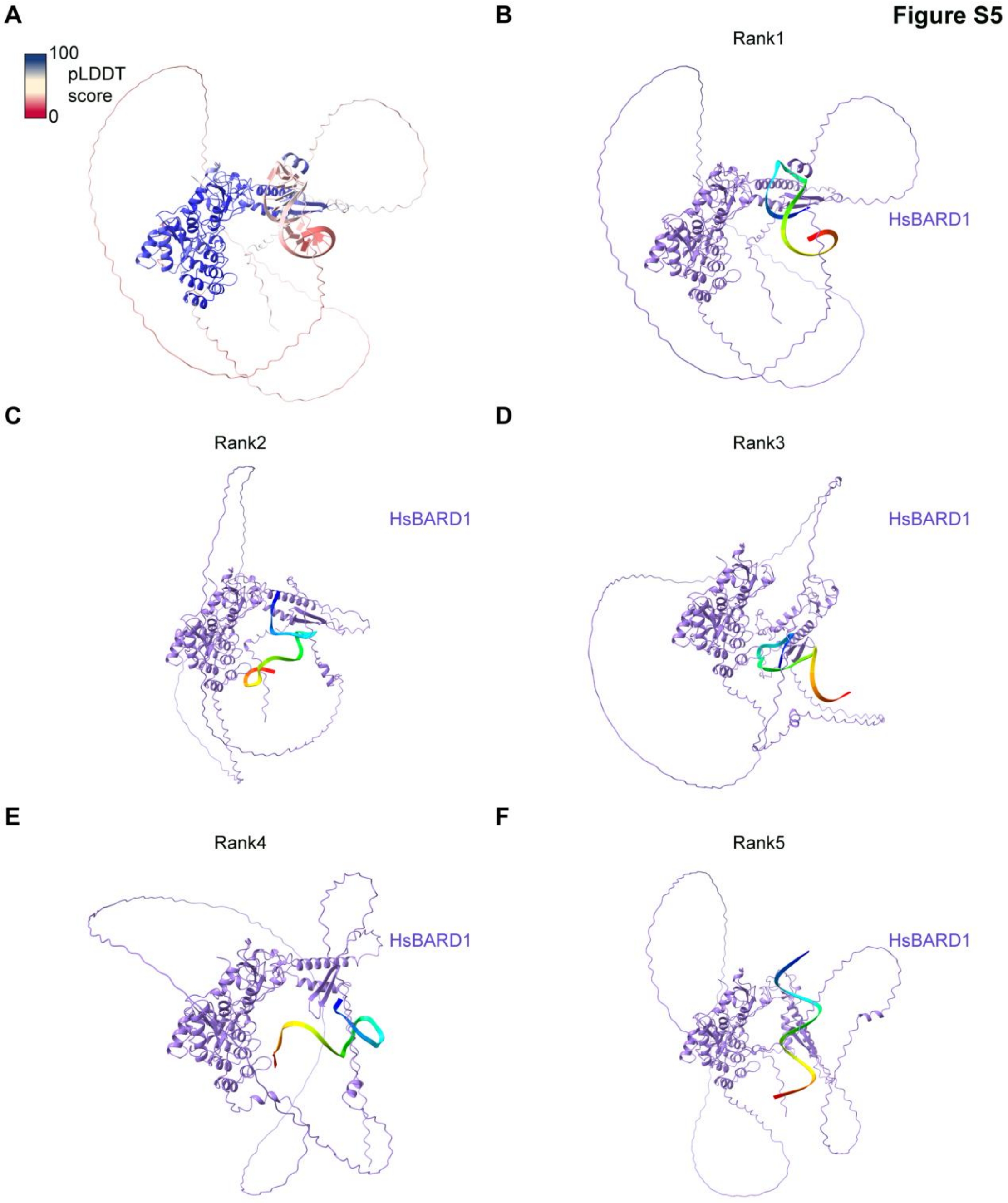

Figure S5. AlphaFold-Multimer analysis of the HsBARD1–pre-rRNA complex.

(A) Human structural model colored by per-residue pLDDT scores (blue: high confidence; red: low confidence). (B–F) The top five structural models predicted by AF3 at a 1:1 stoichiometry. Consistent with the *C. elegans* models, the pairwise RMSD is below 1.0 Å, and structural variance is limited to disordered regions.

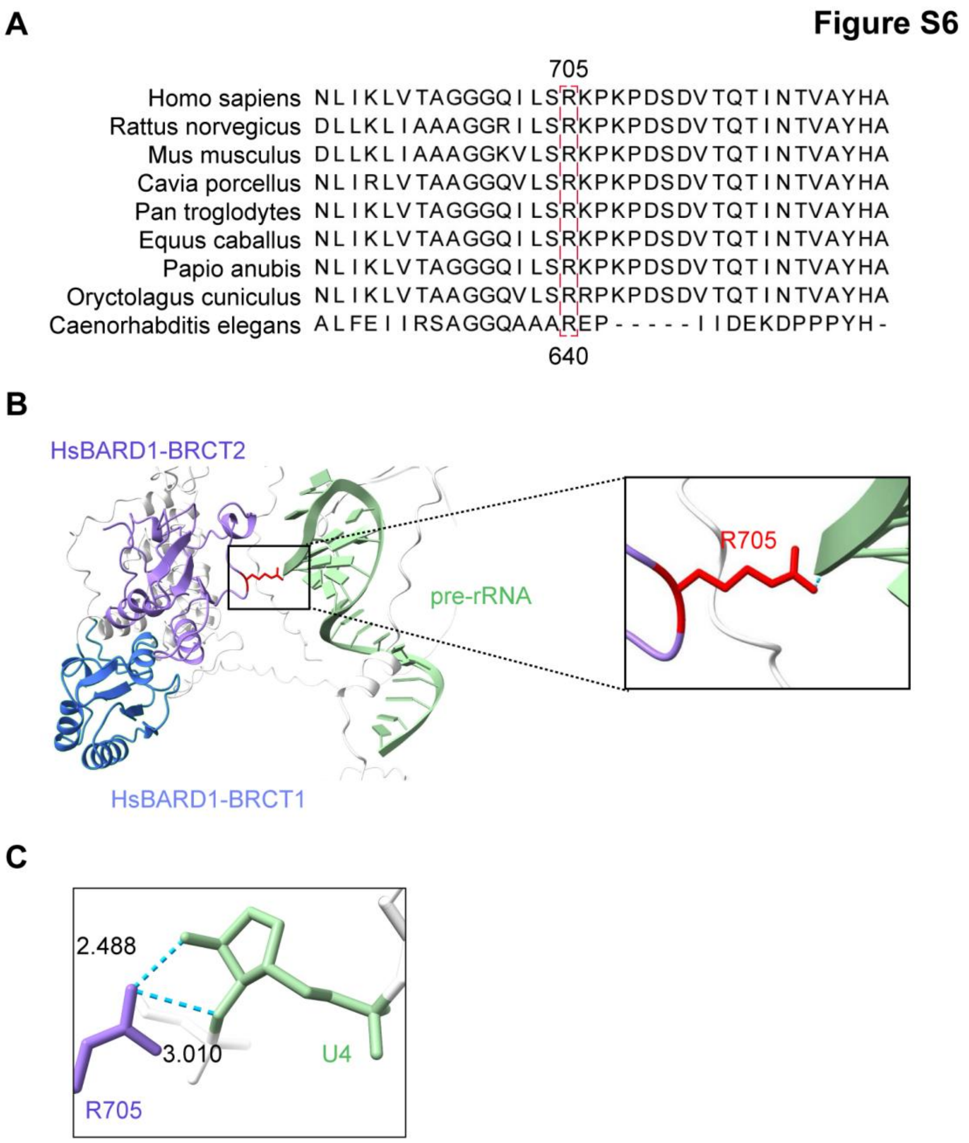

Figure S6. R705 as a conserved functional anchor in the Human BARD1 BRCT domain.

(A) Sequence alignment of human BARD1 (residues 690–724) with indicated orthologues. The red dashed box denotes the highly conserved RNA-binding residue. (B–C) AF3-based structural models identifying R705 as a critical residue for the BARD1–pre-rRNA interaction. The tandem BRCT domains are rendered with BRCT1 in blue and BRCT2 in purple. Hydrogen bonds are indicated by blue dashes, with distances (Å) specified.

Figure S7

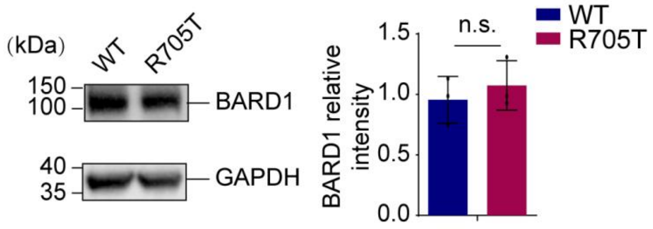

Figure S7. Verification of WT and R705T BARD1 reconstitution levels.

Immunoblot analysis confirmed comparable expression levels of SFB-tagged WT and R705T mutant BARD1 in 293T cells following CRISPR/Cas9-mediated knockout of endogenous BARD1. Data are shown as mean ± SD (n = 3).

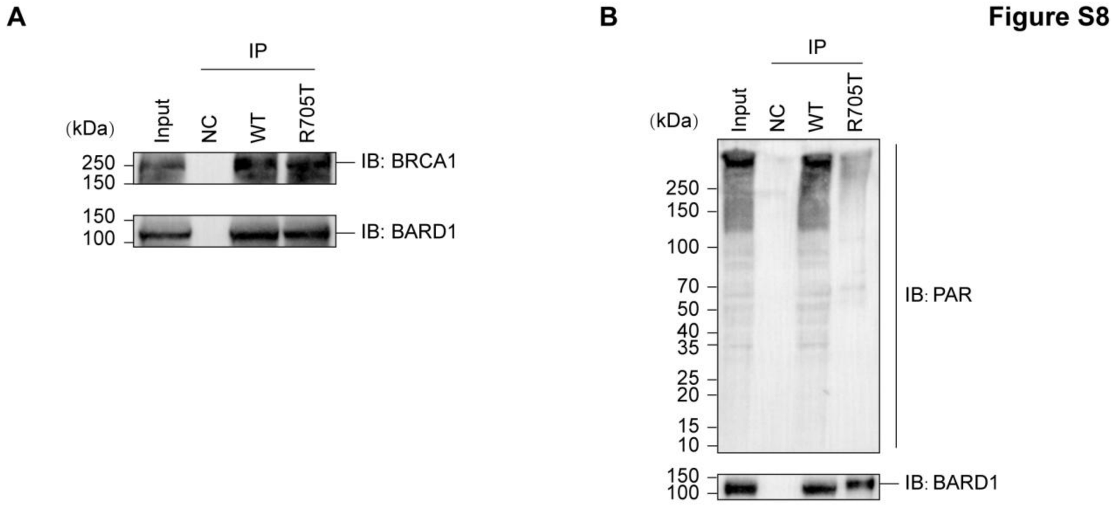

Figure S8. The R705T mutation selectively disrupts nucleic acid binding while maintaining protein-protein interactions.

(A) Co-IP analysis demonstrating that the R705T mutation does not impair the interaction between BARD1 and BRCA1. (B) Conversely, the R705T mutation significantly abrogates BARD1 binding to poly (ADP-ribose) (PAR). Experiments were performed using SFB-tagged constructs in 293T cells, with a negative control (NC) included.

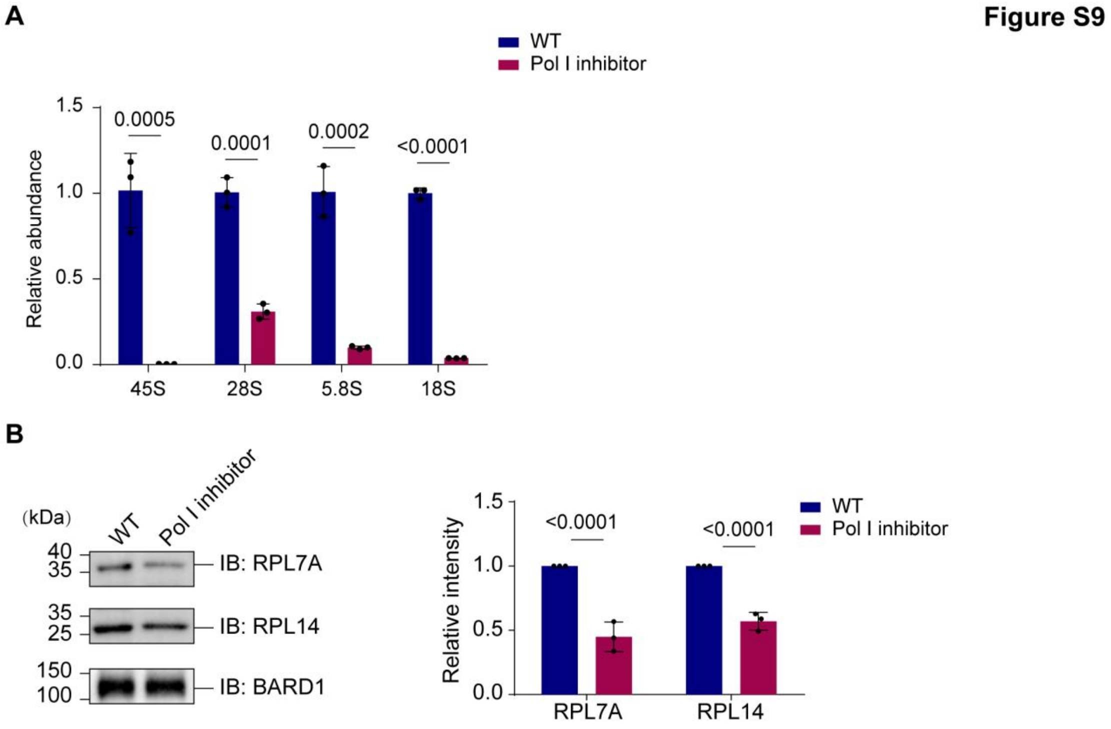

Figure S9. Pol I activity is required for BARD1–pre-rRNA and BARD1–ribosomal protein associations.

(A) Inhibition of Pol I abolishes the association between BARD1 and pre-rRNA, as quantified by Co-IP and RT-qPCR. (B) Pol I inhibition disrupts interactions between BARD1 and ribosomal protein subunits, analyzed via Co-IP and immunoblotting. Data are shown as mean ± SD (n = 3).

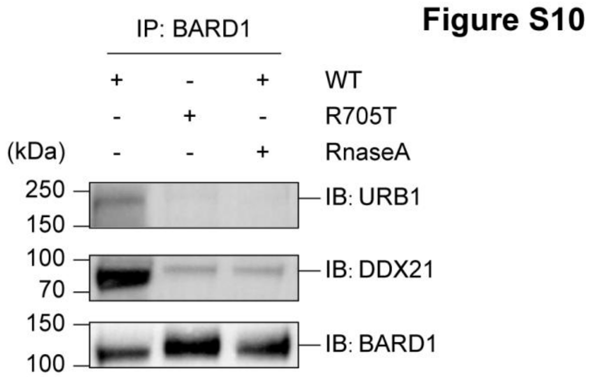

Figure S10. Assembly of the BARD1–PDFC complex is RNA-dependent.

The interaction between BARD1 and PDFC markers is abrogated following either RNase A treatment or the introduction of the R705T mutation. Analysis was conducted via Co-IP and immunoblotting in 293T cells.

Figure S11

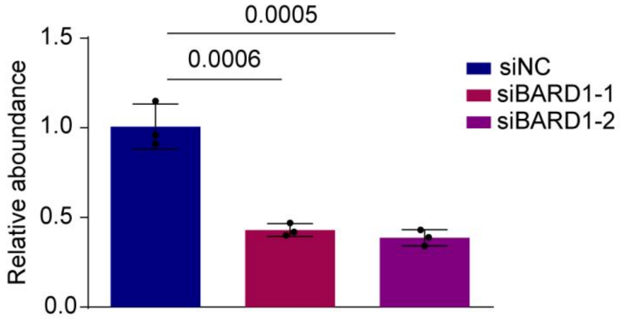

Figure S11. Verification of siRNA-mediated *BARD1* knockdown.

RT-qPCR analysis confirming the successful reduction of *BARD1* mRNA levels in 293T cells treated with targeted siRNAs. Data are shown as mean ± SD (n = 3).

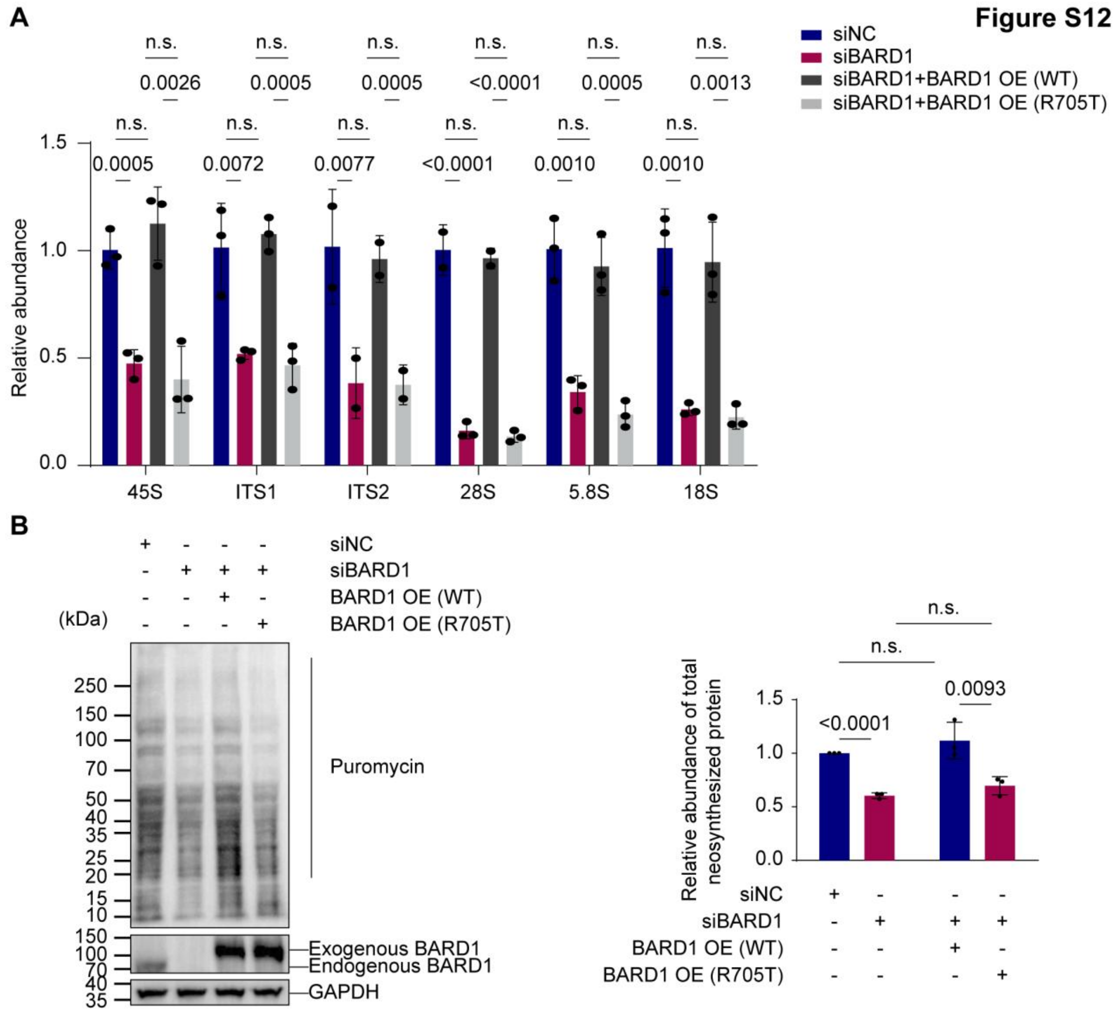

Figure S12. BARD1-mediated rescue of rRNA biogenesis and translation is dependent on the R705 interface.

(A) Pre-rRNA biogenesis (including precursor, intermediate, and mature species) is rescued by the expression of WT BARD1 but not the R705T mutant in HCT116 cells. (B) Global protein synthesis, measured by puromycin incorporation, is similarly rescued only by WT BARD1. Data are shown as mean ± SD (n = 3).

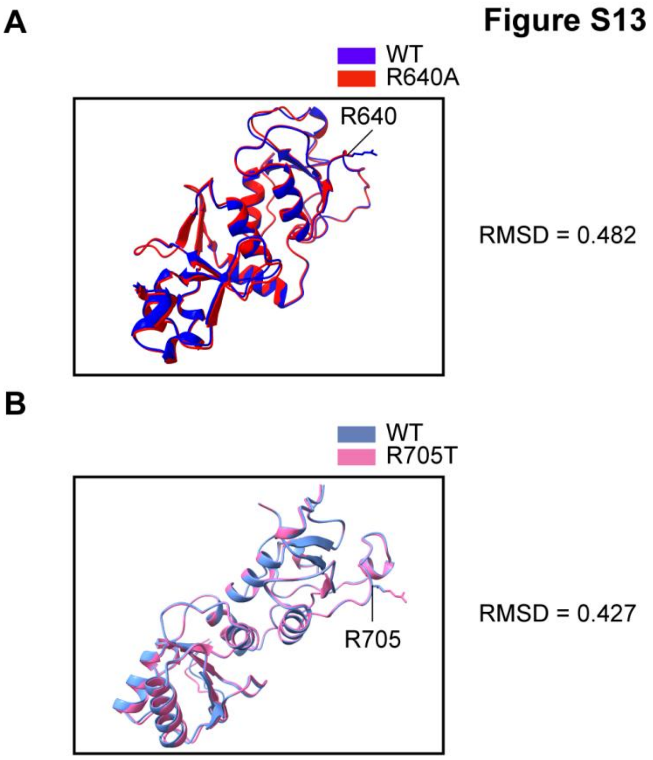

Figure S13. Structural integrity of RNA-binding deficient BARD1 mutants.

(A–B) AF3 structural comparisons confirming that the R640A mutation in *C. elegans* (A) and the R705T mutation in humans (B) do not disrupt the overall BRCT domain fold or induce significant conformational changes. RMSD values (Å) were determined using UCSF ChimeraX.

Table S1. Pairwise RMSD values (Å) for the top five CeBCBD–pre-rRNA structural models.

	Rank1	Rank2	Rank3	Rank4	Rank5
Rank1		0.749	0.619	0.729	0.925
Rank2	0.749		0.675	0.621	0.801
Rank3	0.619	0.675		0.602	0.620
Rank4	0.729	0.621	0.602		0.767
Rank5	0.925	0.801	0.620	0.767	

Table S2. Pairwise RMSD values (Å) for the top five HsBARD1–pre-rRNA structural models.

	Rank1	Rank2	Rank3	Rank4	Rank5
Rank1		0.467	0.534	0.592	0.598
Rank2	0.467		0.354	0.306	0.471
Rank3	0.534	0.354		0.495	0.413
Rank4	0.592	0.306	0.495		0.496
Rank5	0.598	0.471	0.413	0.496	

Table S3. Primers used for the construction of mutant plasmids.

Primer Name	Sequence
CeBARD1 K615A-F	CATTCCGgcgCCGAAGTACAACTTTCTGGACCG
CeBARD1 K615A-R	ACTTCGGcgcCGGAATGAAGAATTTGCAGCCCG
CeBARD1 P624A-F	GGACCGTgctGCGCTCTTCGAGATCATACGTTC
CeBARD1 P624A-R	AGAGCGCagcACGGTCCAGAAAGTTGTACTTCG
CeBARD1 R640A-F	CGCGGCAgctGAACCGATTATCGATGAGAAAGATCC
CeBARD1 R640A-R	TCGGTTCagcTGCCGCGGCTTGTCCGCCCGCGGAAC
HsBARD1 R705T-F	CCTCAGTAcAAAGCCCAAGCCAGACAGTGACG
HsBARD1 R705T-R	TGGGCTTTgTACTGAGGATCTGGCCCCCACCT

Table S4. Primers used for qPCR.

Primer Name	Sequence
45S-qPCR-F	CCCACCCTCGGTGAGAAAAG
45S-qPCR-R	GGAAGCGGAGGAGGGTCCTC
18S-qPCR-F	GGCCCGAAGCGTTTACTTTG
18S-qPCR-R	GCGGCGCAATACGAATGCC
28S-qPCR-F	CACGAGACCGATAGTCAACAAG
28S-qPCR-R	AACGGGGGGCGGGAAAGATC
5.8S-qPCR-F	GACTCTTAGCGGTGGATCAC
5.8S-qPCR-R	AAGCGACGCTCAGACAGGC
ITS1-qPCR-F	CGAGAGCCGGAGAACTCGG
ITS1-qPCR-R	GCCGACACCCACGTCGTC
ITS2-qPCR-F	CGGGCCCTGCGTGGTCAC
ITS2-qPCR-R	GGAGGAACCCGGACCGCAG
GAPDH-F	GTCTCCTCTGACTTCAACAGCG
GAPDH-R	ACCACCCTGTTGCTGTAGCCAA

Table S5. Synonymous mutation sites designed in human BARD1.

CDS Mutation	AA Mutation
267G>A	Pro89Pro
741T>A	Ser247Ser
1131G>A	Gly377Gly
1467C>A	Thr489Thr

Table S6. Sequences of oligos for sgRNA.

Oligo name	Sequence
sgRNA1-S	CACCGGTAAAGTTGGTGGTACATCA
sgRNA1-AS	AAACTGATGTACCACCAACTTTACC
sgRNA2-S	CACCGAAGGCATTGGTGAACACCAC
sgRNA2-AS	AAACGTGGTGTTCACCAATGCCTTC
sgRNA3-S	CACCGATGTCCAGTGTGTTACACCC
sgRNA3-AS	AAACGGGTGTAACACACTGGACATC
sgRNA4-S	CACCGTGAGGACTGGAGATAACAGA
sgRNA4-AS	AAACTCTGTTATCTCCAGTCCTCAC

Table S7. Sequences of oligos for siRNA.

Oligo name	Sequence
siBARD1-1-S	GAAAUAGACUUACUAGCAA
siBARD1-1-AS	UUGCUAGUAAGUCUAUUUC
siBARD1-2-S	GUAAGUGACUGCAUUGGAA
siBARD1-2-AS	UUCCAAUGCAGUCACUUAC
